# Supplementary material for: Mechanism for Collective Cell Alignment in Myxococcus xanthus Bacteria
Source: PLoS Comput Biol. 2015 Aug 26;11(8):e1004474. doi: 10.1371/journal.pcbi.1004474 (PMC4550276; doi:10.1371/journal.pcbi.1004474)
Supplement: S1 Text — Details of quantification procedures for cell clustering along with the simulation parameters. (DOCX) [file pcbi.1004474.s001.docx]

TextS1: **Quantitative measurements of cell clustering**

**Cluster size distribution (CSD)**

We identify the clusters in the simulation region using a density based clustering algorithm (DBSCAN [SR, SR6]) applied on agent node positions. This algorithm identifies groups of nodes that exceed given density threshold and classifies them as separate clusters. We chose the parameters of the algorithm (minimum number of nodes to form a cluster, $k=21$ i.e., 3 cells, and the minimum neighbor distance, $d_{min}=2$) that resulted in good separation (visually) between individual clusters. Using small neighbor distance ($d_{min}\leq2$) values in this algorithm resulted in large clusters that are actually multiple separate clusters connected by a narrow streams of agents. So we used minimum neighbor distance as $d_{min}=2 \mu m$ and later processed the individual clusters to include short distance ($\leq2 \mu m$) neighbor agents.

Next, we determine the agents belonging to each separate cluster of nodes identified by the algorithm. We process partial agents i.e., agents for which only fraction of their nodes are included into a cluster, to include all their nodes into the cluster. We further process the clusters to include all the nearest neighbor agents ($d_{min}<0.75 \mu m$) that are missed by the algorithm. We quantify the size of the clusters ($m$) by measuring the number of agents in each cluster. Snapshots of identified clusters (after processing) from simulation are shown in M1Fig.


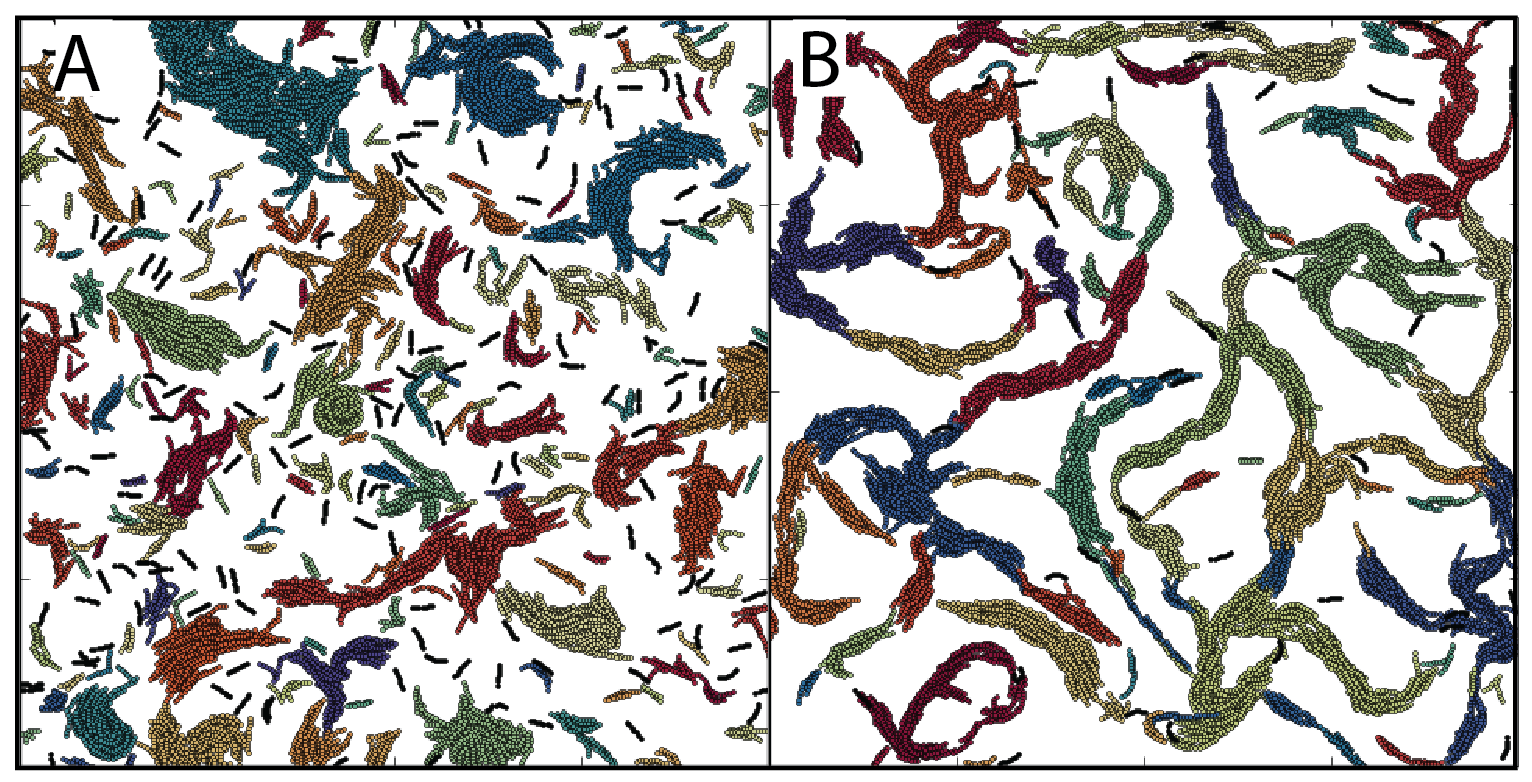


**Fig.** **M1 Identifying cell clusters using DBSCAN algorithm** Different colors represent different clusters of agent nodes identified from their positions that exceed given density threshold. Snapshots of identified clusters for (A) Non-reversing cells without slime-trails (B) Reversing cells with slime-trail-following mechanism ($L_{s}=11 \mu m,\varepsilon_{s}=1.0$) at 180 mins after the simulation started. Cell density$\eta=0.24$

We quantify the cluster size distribution (CSD) by measuring the probability $p(m)$ of finding a cell in a cluster of size $m$. For this, we follow the procedure illustrated in Starruẞ et al.[16]. After identifying and processing the clusters from simulation, we obtain an array of various cluster sizes ($m_{i}$) at each time frame. These values ($m_{i}$) are converted into a normalized histogram ($f_{i}$) whose bin edges are chosen exponentially i.e., bin $i (=1 ,2 , ...)$ contains all agents that belong to clusters of size $10^{(i-1)dm}\leq m<10^{i dm}$, where $dm=0.33$. Thus $f_{i}$ values represent fraction of all agents found in cluster sizes represented by bin $i$. Finally, probabilities, $p_{i}$, are calculated by dividing the $f_{i}$ values with the corresponding bin width $db_{i}\left(=10^{i dm}-10^{(i-1)dm}\right)$.

**Mean cluster size,** $\left\langle\mathbf{m} \right\rangle$

Due to the sparse nature of cluster size distribution data from simulation, we calculate mean cluster size at each cell density ($\eta$) using data from multiple simulation runs and from multiple time points ($\tau_{ss,t}$) in each simulation run after CSD values reached steady state. We chose time points ($\tau_{ss,t}$) such that the CSD at these time points are sufficiently independent. For this we measure the auto-correlation between snapshots of cluster images from simulation as a function of time (M2 Fig). Image auto-correlation is calculated using Eqn. 1 where $C_{I}(t, t+\delta t)$ is the normalized cross correlation between snapshots $(f,g)$ of the simulation at times $t$ and $t+\delta t$, $N_{K}$ is number of such image pairs. Normalized cross correlation between two images $f,g$ is calculated using Eqn. 2 where $n$ is the total number of pixels in the image, $f_{i,j}$ is grayscale intensity of pixel at position $i,j$ in image$f$, and $\bar{f}, \sigma_{f}$ are the average intensity and standard deviation in intensity of pixels in image $f$.

$$C(\delta t)=\frac{1}{N_{K}}\sum_{t=1}^{K} C_{I}(t, t+\delta t)$$

$$C_{I}=\frac{1}{n}\sum_{i,j} \frac{\left(f_{i,j}-\bar{f}\right)\left(g_{i,j}-\bar{g}\right)}{\sigma_{f}\sigma_{g}}$$

Auto-correlation values are measured for snapshots of simulation between 60 to 180 mins after initialization. From these results, we determined that correlation among cluster images dropped to low value (< 0.1) after 20 mins for both reversing and non-reversing cells (M2 Fig). Thus we take data from steady state time points ($\tau_{ss,t}$) separated by 20 mins as independent trials.


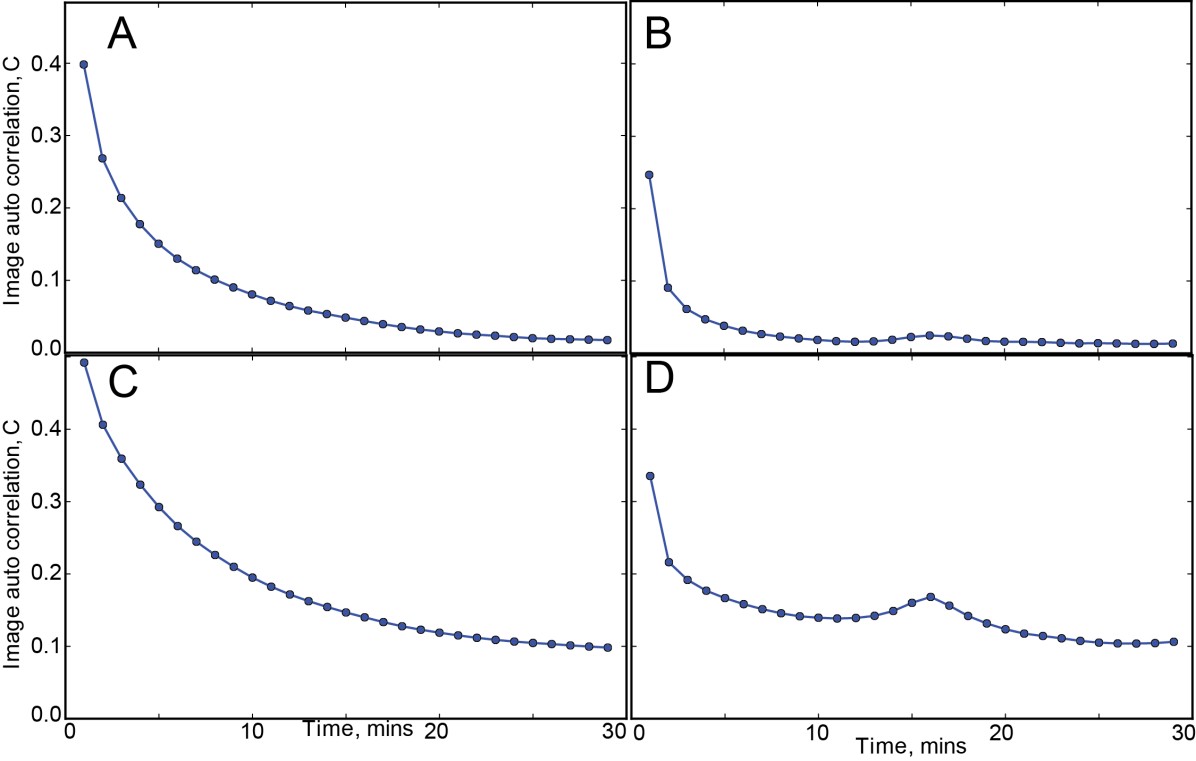


**M2 Fig. Auto-correlation of cluster images with time**. Simulations with (A, C) non-reversing cells, (B, D) reversing cells. (C, D) Simulations with slime-trail-following mechanism with (C) model parameters $L_{s}=0.6 \mu m, \varepsilon_{s}=0.5$ and (D) model parameters $L_{s}=11 \mu m, \varepsilon_{s}=0.2$. All these simulations are performed at cell density $\eta=0.24$. Correlation values are computed for snapshot images from 60 - 180 mins of the simulation time. Correlation among simulation images dropped to low value ($<0.1$) after 20 mins.

Mean cluster sizes at each time point ($\tau_{ss,t}$) is calculated using $\bar{m}_{t}=\sum_{i} p_{i}m_{i}$, where $p_{i}$ is the probability from CSD and $m_{i}$ is the average cluster size ($=10^{(i-1)dm}+(10^{(i-1) dm}-10^{i dm})/2$) of bin $i$. Finally the mean $\left\langle m\right\rangle$ and standard deviation in cluster sizes at each cell density is calculated by averaging data from all the steady state time points from multiple simulation runs ($n=5$).

**Orientation correlation,** $C(r)$

Alignment among neighbor agents is quantified using orientation correlation function $C(r)=\left\langle\cos2\Delta\theta_{r}\right\rangle$. Here $\Delta\theta_{r}$ is the angle deviation between orientations of a pair of neighbor cells whose center nodes are separated by a distance $r$ (S7F Fig). represents average over all cell pairs that are separated by a distance $r$.

**M1 Table.**  **Parameters used in simulation**

| **Symbol** | **Description** | **Value** |
| --- | --- | --- |
| $L_{sim}$ | Dimension of square simulation region | $200 \mu m$ |
| $M$ ($\eta$) | Total number of agents  (corresponding cell densities ) | 246-3938  ($0.02-0.32$) |
| $\varepsilon_{s}$ | Slime effectiveness factor | 1.0 |
| $L_{s}$ | Slime-trail length | $11 \mu m$ |
| $k_{d}$ | Slime degradation constant | $1.0 1/\min$ |
| $V_{s}$ | Slime production rate | 20 AU |
| $L_{c}$ | Agent length | $6.5 \mu m$ [6, SR3] |
| $W_{c}$ | Agent width | $0.5 \mu m$ [6, SR3] |
| $k_{b}$ | Angular spring constant/bending stiffness | $10^{-17} Nm$ [SR3-5] |
| $v_{c}$ | Mean speed of cell | $4 \mu m/\min$ [41] |
| $t_{step}$ | Simulation time step | $5\times10^{-3}\min$ |
| $k_{a}$ | Spring constant for cell-substrate adhesions | $100 pN/\mu m$[20] |
| $F_{T}$ | Propulsive force per cell | 60 pN [20] |
| $\tau_{r}$ | Reversal period | 8 min [4] |

**Supplemental References**

SR1. Ester M, Kriegel H-P, Sander J, Xu X. A density-based algorithm for discovering clusters in large spatial databases with noise 1996.

SR2. Pedregosa F, Varoquaux G, Gramfort A, Michel V, Thirion B, et al. (2011) Scikit-learn: Machine Learning in Python. J Mach Learn Res 12: 2825-2830.

SR3. Janulevicius A, van Loosdrecht MC, Simone A, Picioreanu C (2010) Cell flexibility affects the alignment of model myxobacteria. Biophysical journal 99: 3129-3138.

SR4. Wolgemuth CW (2005) Force and flexibility of flailing myxobacteria. Biophysical journal 89: 945-950.

SR5. Harvey CW, Morcos F, Sweet CR, Kaiser D, Chatterjee S, et al. (2011) Study of elastic collisions of Myxococcus xanthus in swarms. Physical biology 8: 026016.
