# Supplementary figures and images for: Mechanism for Collective Cell Alignment in Myxococcus xanthus Bacteria
Source: PLoS Comput Biol. 2015 Aug 26;11(8):e1004474. doi: 10.1371/journal.pcbi.1004474 (PMC4550276; doi:10.1371/journal.pcbi.1004474)

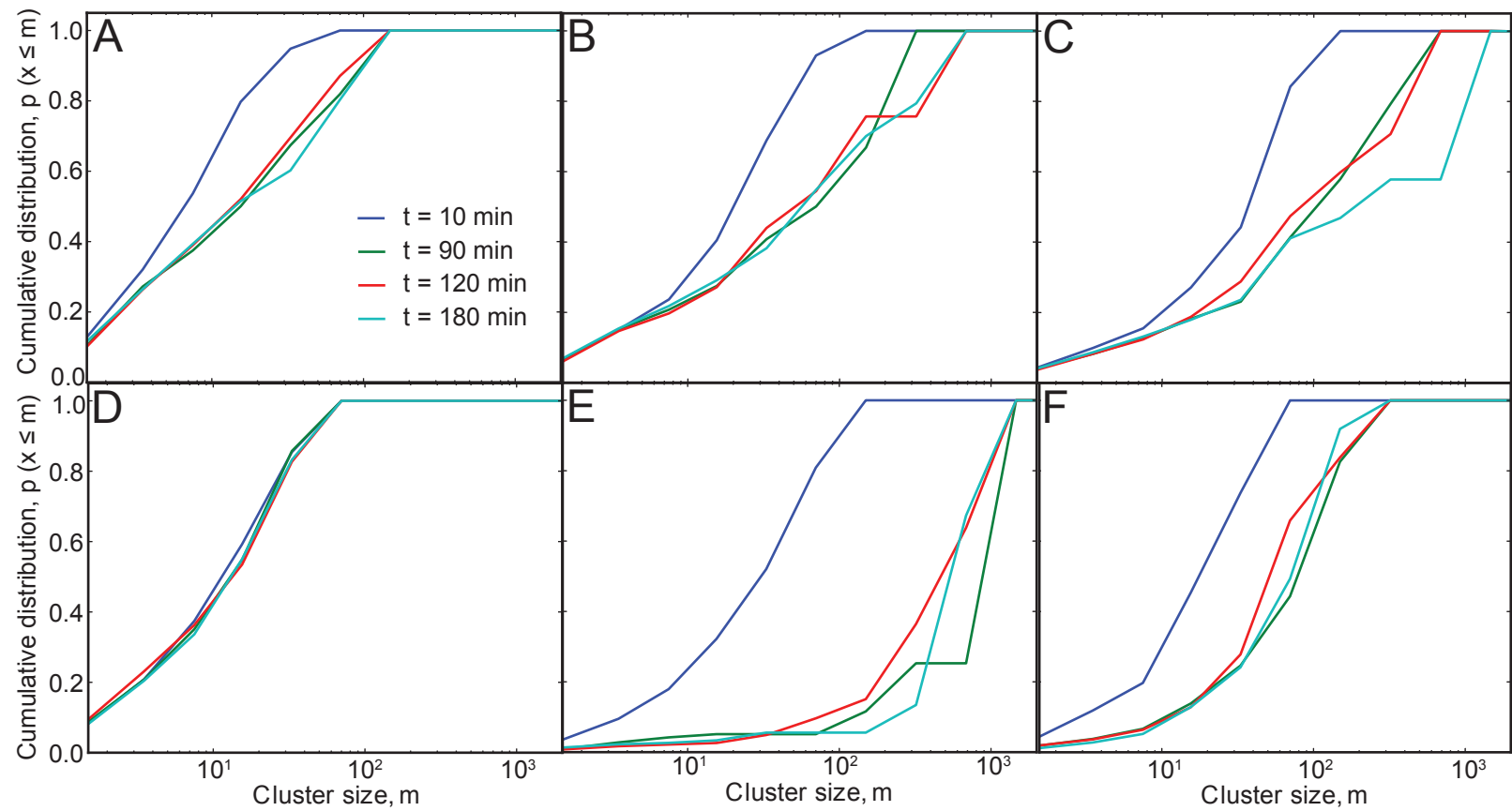

Supplement: S1 Fig — Non-reversing agents with cell densities (A) η = 0.08 (B) η = 0.16 (C) η = 0.24 (D) η = 0.32 (E) Reversing agents with cell density η = 0.24 (F) Non-reversing agents following slime-trails, η = 0.24 (G) Reversing agents following slime-trails, η = 0.24. (PDF) [file pcbi.1004474.s002.pdf]

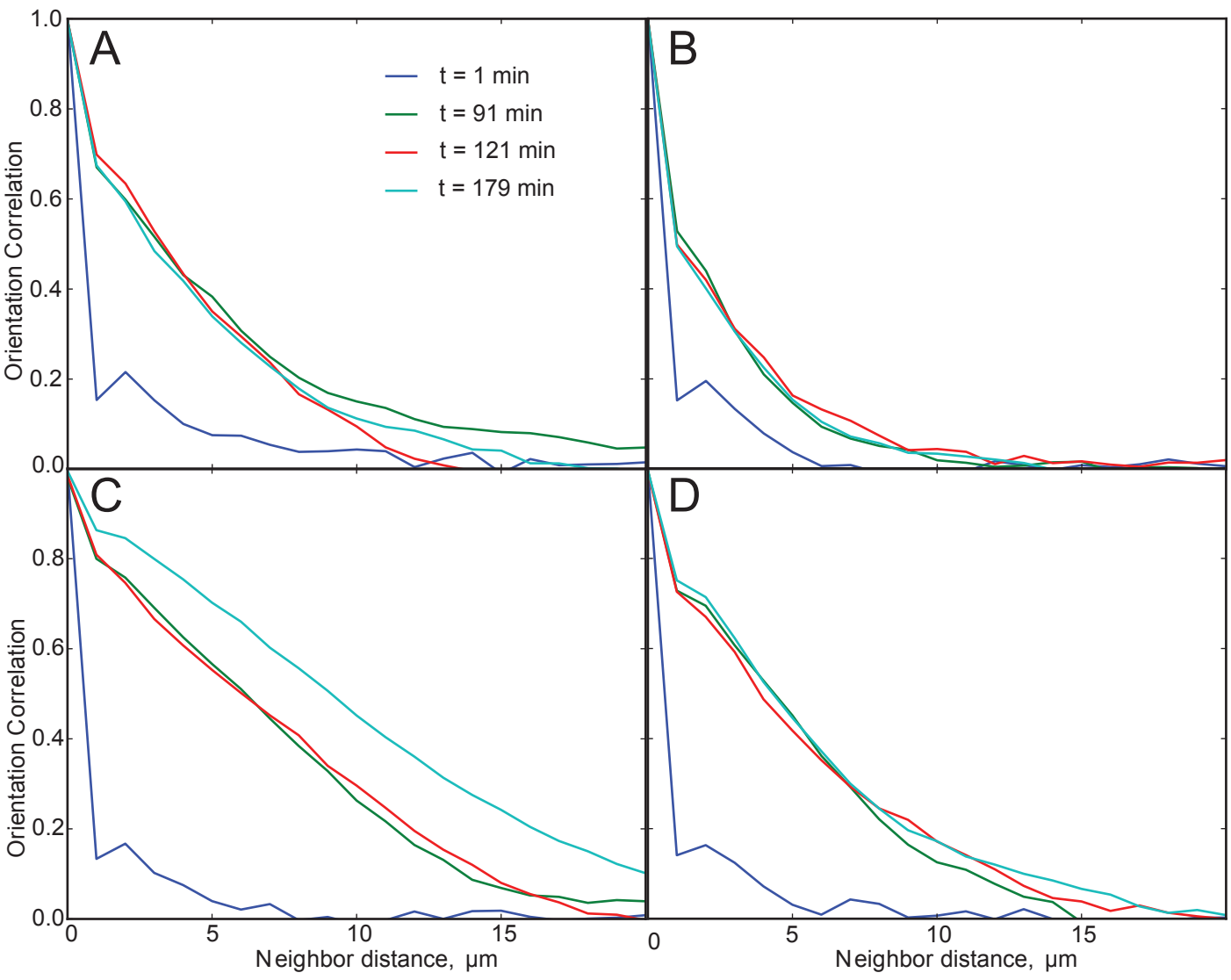

Supplement: S2 Fig — (A, C) Non-reversing cells (B, D) Reversing cells (C, D) cells following slime-trails. All simulations performed at cell density η = 0.24. (PDF) [file pcbi.1004474.s003.pdf]

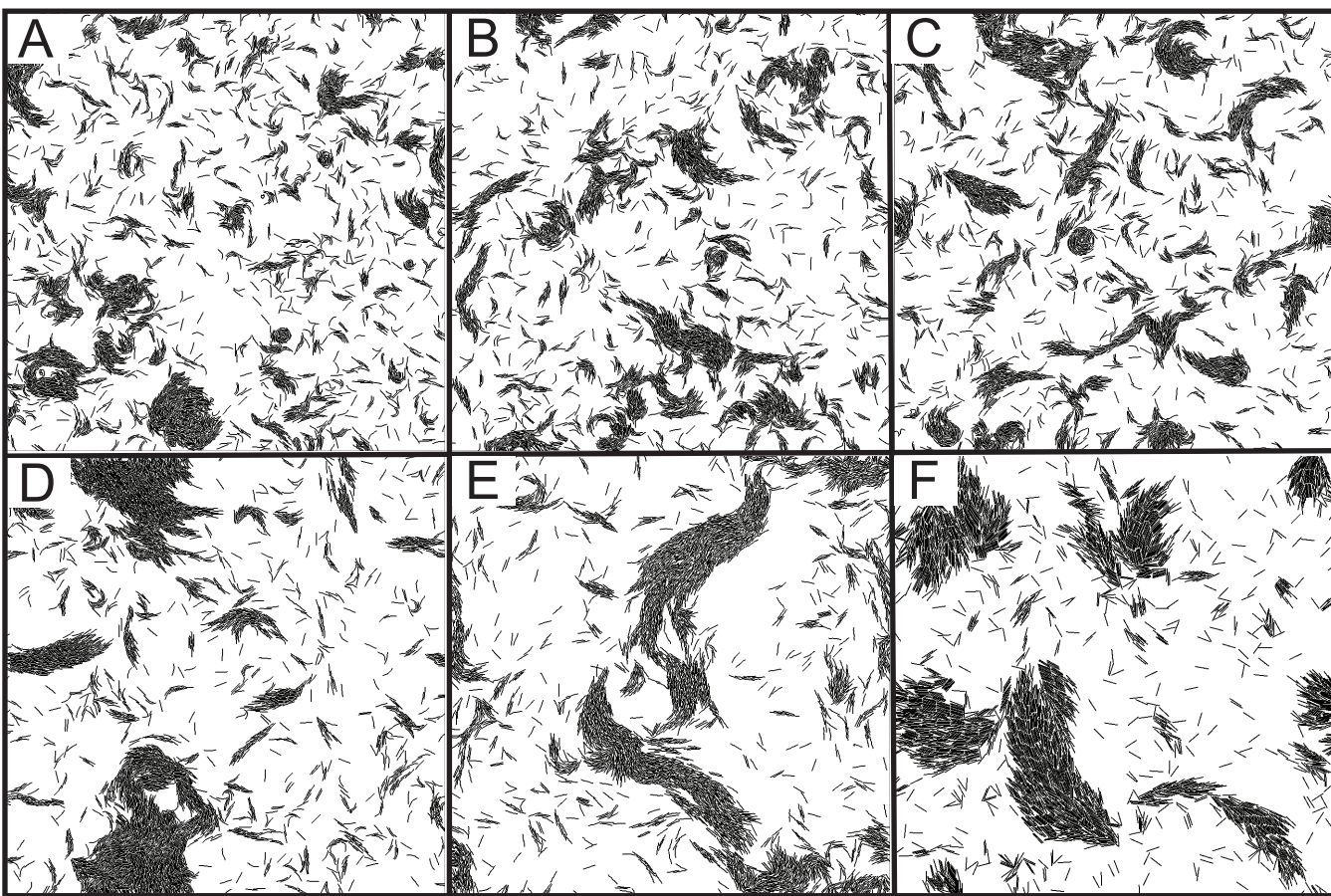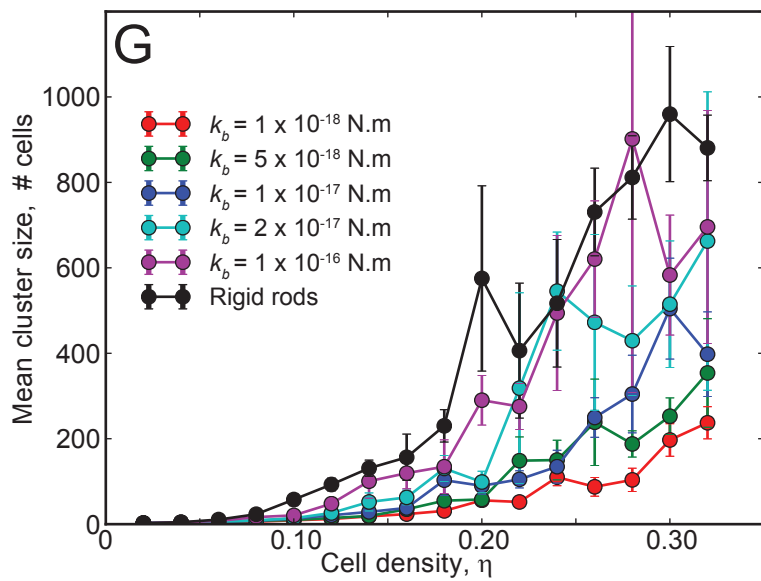

Supplement: S3 Fig — (A-F) Snapshots of cell clusters after 180 min of simulation (cell density, η = 0.24) with bending stiffness (k b) values (A) 10−18 N.m (B) 5 × 10^−18^ N.m (C) 10−17 N.m (D) 2 × 10^−17^ N.m (E) 10−16 N.m (F) Rigid rods (G) Mean cluster sizes in simulation as a function of cell density (η) for different cell bending stiffness values. (PDF) [file pcbi.1004474.s004.pdf]

**A**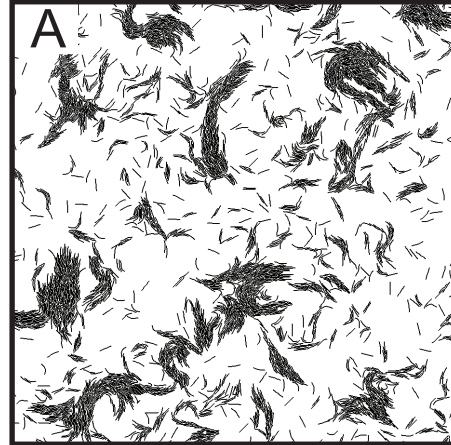**B**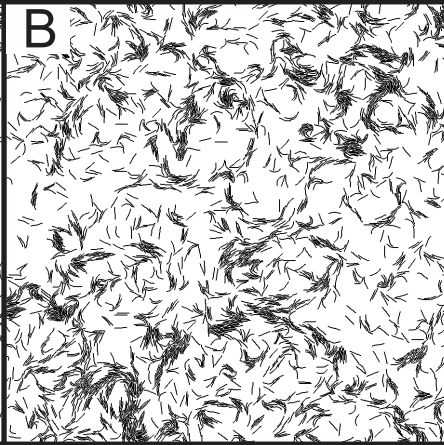**C**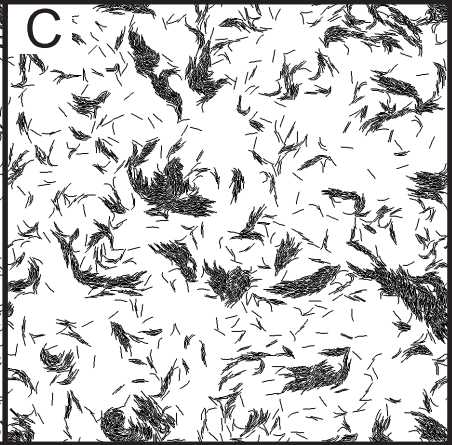

Supplement: S4 Fig — Snapshots of simulation at (A) 90 min (B) 120 min (C) 150 min. Cell reversals were turned-off for the first 90 min of simulation and thereafter are turned-on from 90 to 120 min with reversal period = 8 min. Reversals are turned-off again at 120 min. Cell clusters formed by simulating non-reversing cells for first 90 min (A) are quickly, within 30 min destroyed by cell reversals (B). Suppression of reversals restored clustering of cells after another 30 min (C). (PDF) [file pcbi.1004474.s005.pdf]

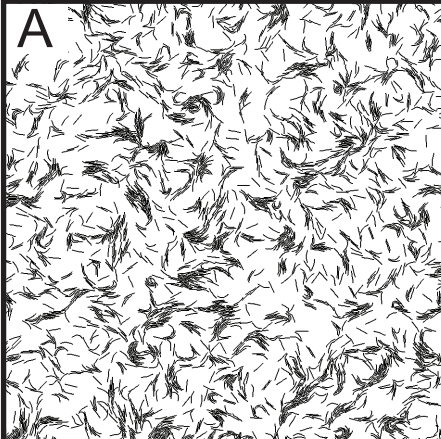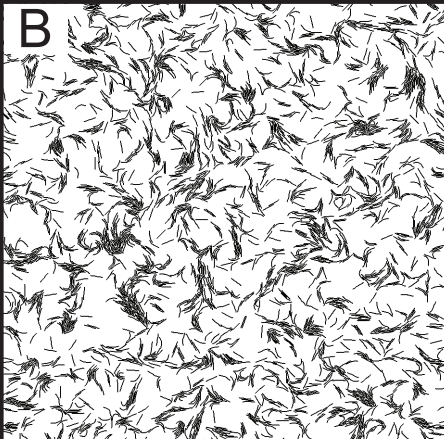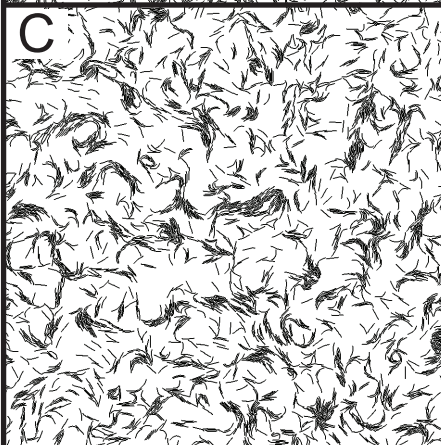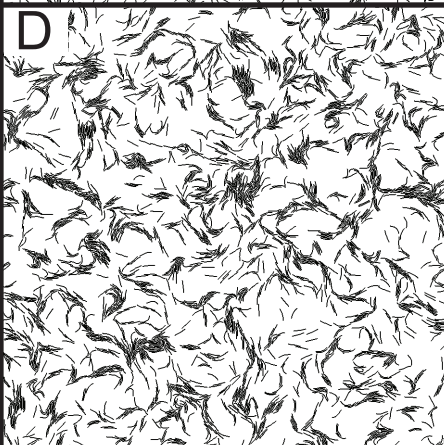

Supplement: S5 Fig — Snapshots of simulation at 180 min for different lateral adhesion force values. Adhesion force per cell (A) F adh = 0 pN (B) F adh = 30 pN (C) F adh = 60 pN (D) F adh = 120 pN. (PDF) [file pcbi.1004474.s006.pdf]

Non-reversing cells

Reversing cells

No slime-trail-following

Slime-trail-following

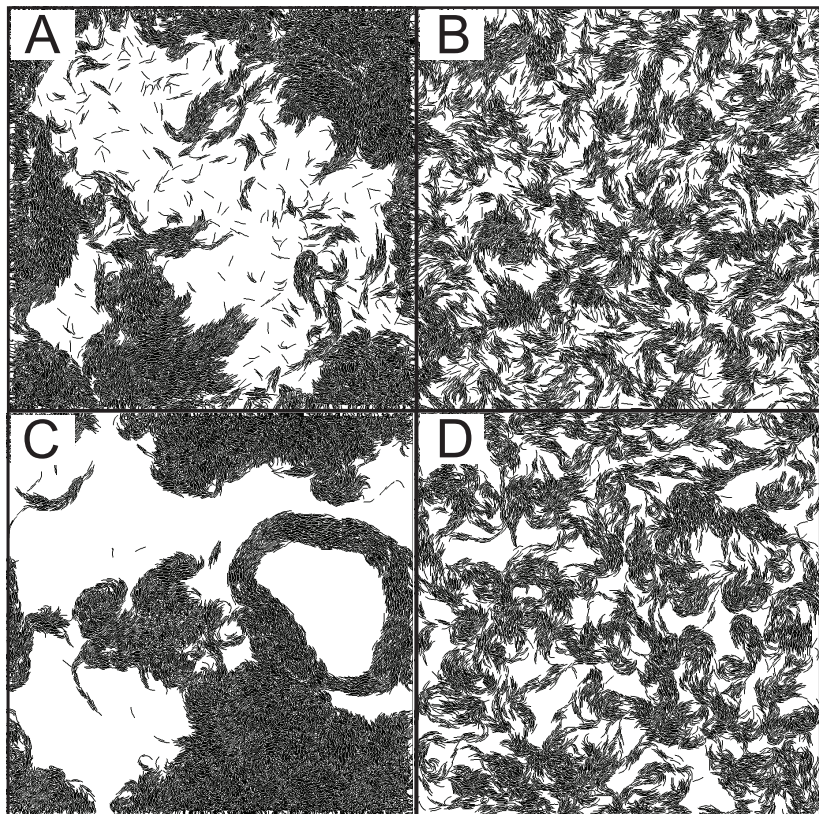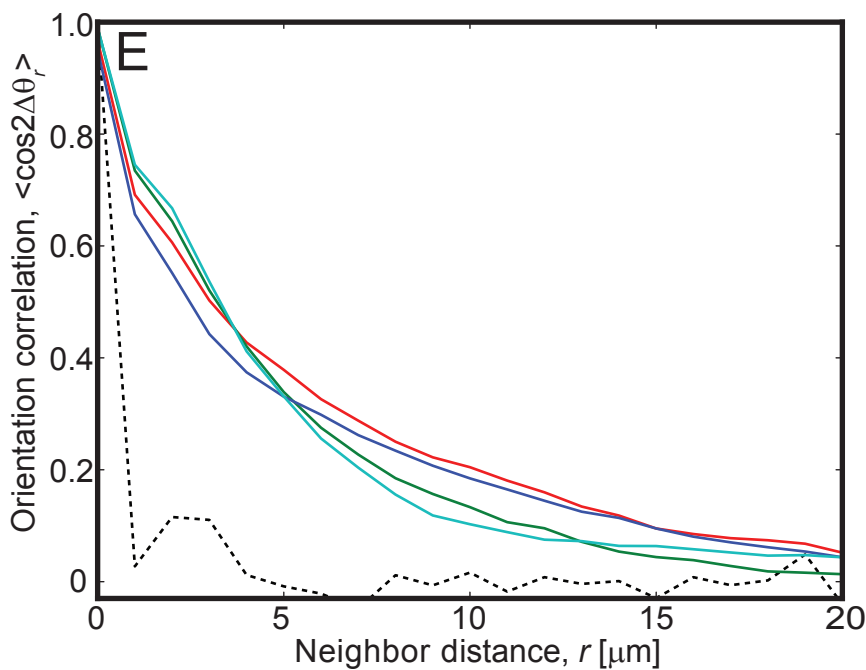

Supplement: S6 Fig — (A-D) Snapshots of simulation at 180 min for cell density η = 0.60. (E) Orientation correlation among cells at 180 min of simulation time for non-reversing cells (red), reversing cells (green), non-reversing cells with slime-trail-following (blue), and reversing cells with slime-trail-following (cyan). Dotted line represents the orientation correlation values at 1 min simulation time. (PDF) [file pcbi.1004474.s007.pdf]

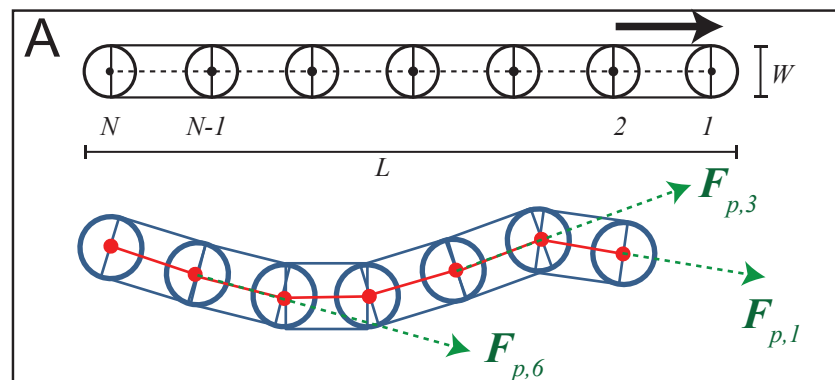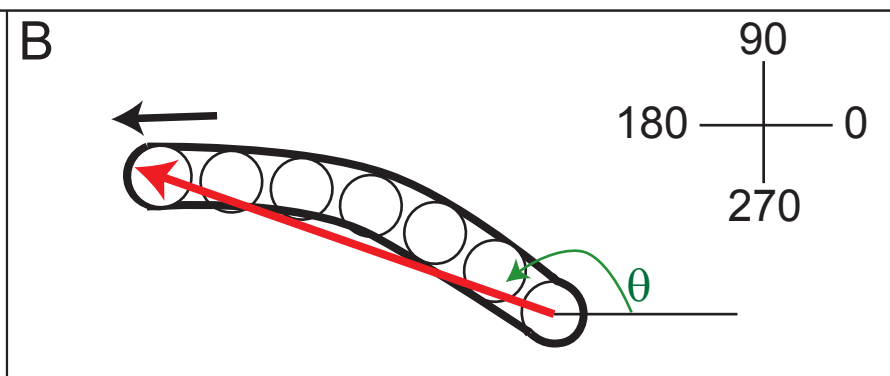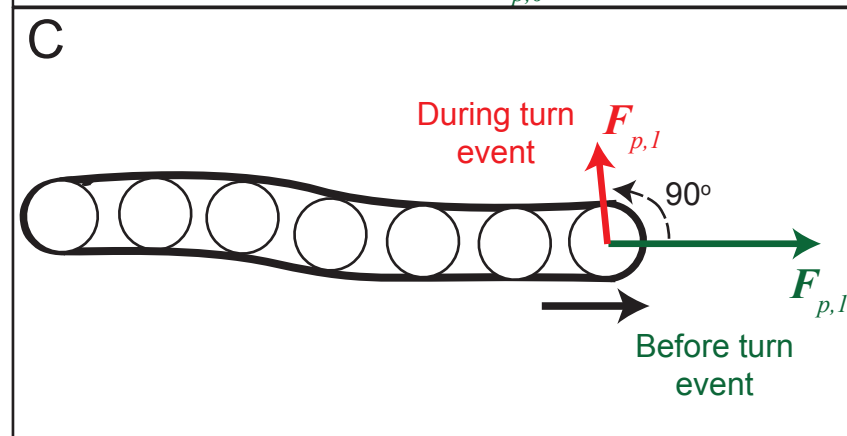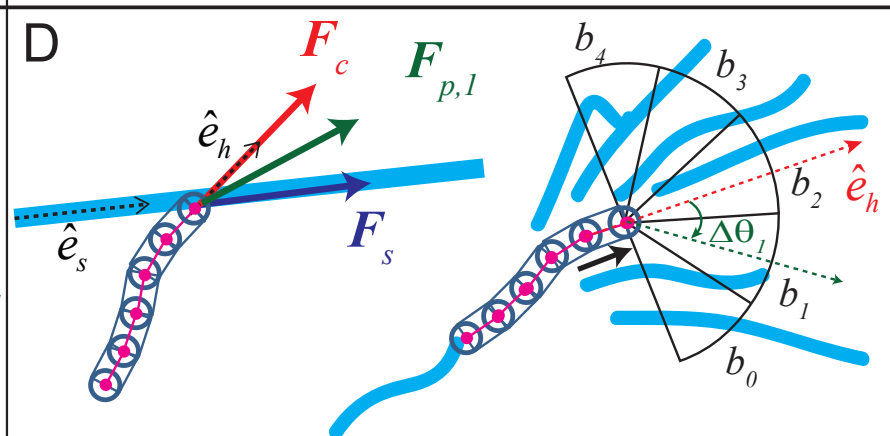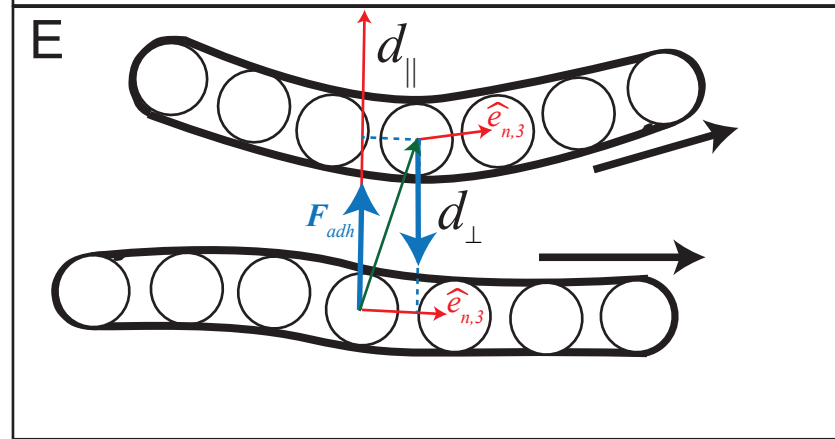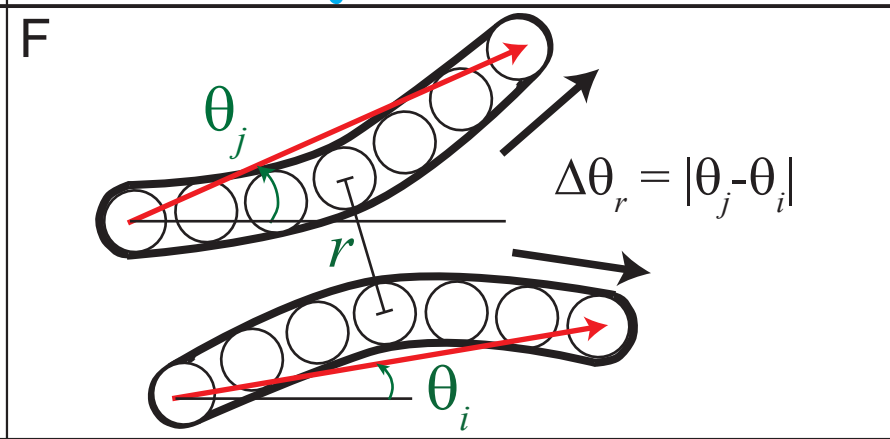

Supplement: S7 Fig — (A) Each agent contains N = 7 nodes connected by joints that simulate elastic behavior of the cell. Propulsive forces (F p,i, green arrows) on the nodes, in the direction of next node, move the agent forward. (B) Orientation (θ) of an agent defined as the angle made by the vector connecting from its tail node to head node with the X-axis. (C) Random noise in agent direction is introduced by reorienting the propulsive force (F p,1) on its head node by 90° either clockwise or anti-clockwise randomly for a fixed amount of time (= 1 min). (D) Schematic for implementation of slime-trail following. When an agent encounters a slime trail, a part of the propulsive force on its head node (F s) proportional to amount of slime in the trail is reoriented parallel to the direction of the slime-trail (e^s). Remaining propulsive force F c (=(FT/(N−1)−|Fs|)e^h) acts in current head node direction (e^h). Thus the resulting force on the head node F p,1 maintains its magnitude but changes its direction due to its interaction with slime. In slime-rich regions (slime denoted by blue trails) of simulation, effective slime-trail direction (e^s) is estimated by dividing a semi-circular slime search region at the head node of the agent into bins (n = 5). e^s is chosen as the direction (center line) of the bin with high slime volume (0.8 S max) but with least deviation (Δθ s) from current head node direction (e^h). (E) Lateral adhesive forces (F adh) between a pair of agents acting normal to node propulsion vectors (e^n,i). These forces are implemented for simulations shown in S5 Fig only (F) Orientation correlation between a pair of agents, is computed by averaging cos(2Δθ r) over all agent pairs whose center nodes are separated by distance r. Δθ r is the difference in orientations between the two agents. (PDF) [file pcbi.1004474.s008.pdf]
