# Supplementary material for: Mechanism for Collective Cell Alignment in Myxococcus xanthus Bacteria
Source: PLoS Comput Biol. 2015 Aug 26;11(8):e1004474. doi: 10.1371/journal.pcbi.1004474 (PMC4550276; doi:10.1371/journal.pcbi.1004474)
Supplement: S8 Fig — (PDF) [file pcbi.1004474.s009.pdf]

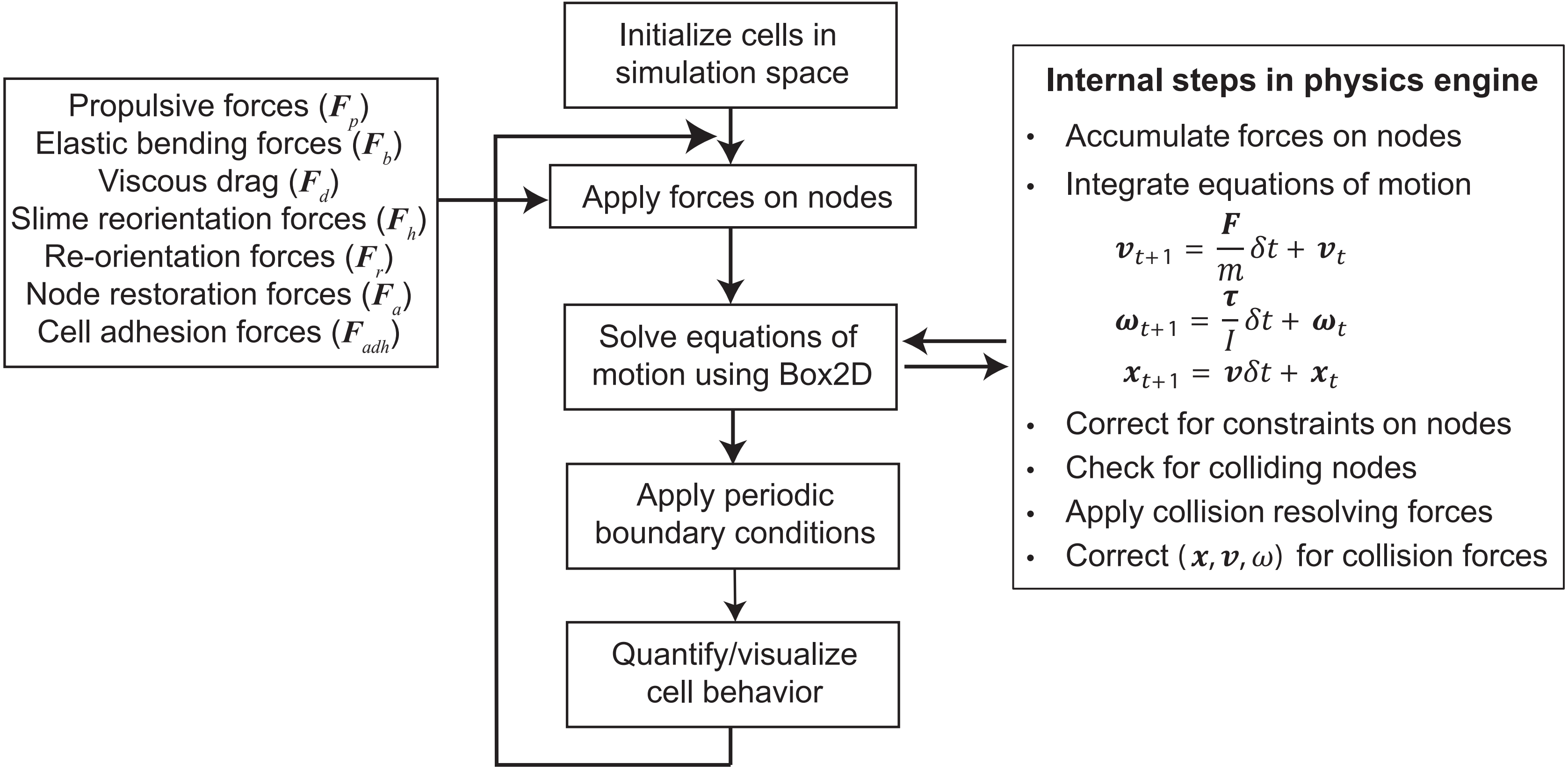
Initialize cells in simulation space
Propulsive forces (F_p)
Elastic bending forces (F_b)
Viscous drag (F_d)
Slime reorientation forces (F_h)
Re-orientation forces (F_r)
Node restoration forces (F_a)
Cell adhesion forces (F_{adh})
Apply forces on nodes
Solve equations of motion using Box2D
Apply periodic boundary conditions
Quantify/visualize cell behavior
Internal steps in physics engine
Accumulate forces on nodes
Integrate equations of motion
$v_{t+1} = \frac{F}{m}\delta t + v_t$
$\omega_{t+1} = \frac{\tau}{I}\delta t + \omega_t$
$x_{t+1} = v\delta t + x_t$
Correct for constraints on nodes
Check for colliding nodes
Apply collision resolving forces
Correct (x, v, ω) for collision forces
